# Zn^2+^ and Cu^2+^ Binding to the Extramembrane Loop of Zrt2, a Zinc Transporter of *Candida albicans*

**DOI:** 10.3390/biom12010121

**Published:** 2022-01-12

**Authors:** Denise Bellotti, Adriana Miller, Magdalena Rowińska-Żyrek, Maurizio Remelli

**Affiliations:** 1 Department of Environmental and Prevention Sciences, University of Ferrara, 44121 Ferrara, Italy; 2 Department of Chemical, Pharmaceutical and Agricultural Sciences, University of Ferrara, 44121 Ferrara, Italy; rmm@unife.it; 3 Faculty of Chemistry, University of Wrocław, 50-383 Wrocław, Poland; adriana.miller@chem.uni.wroc.pl (A.M.); magdalena.rowinska-zyrek@chem.uni.wroc.pl (M.R.-Ż.)

**Keywords:** metal-binding protein, zinc transporter, coordination chemistry, solution equilibria, zinc(II), copper(II), candida albicans, Zrt2

## Abstract

Zrt2 is a zinc transporter of the ZIP family. It is predicted to be located in the plasma membrane and it is essential for *Candida albicans* zinc uptake and growth at acidic pH. Zrt2 from *C. albicans* is composed of 370 amino acids and contains eight putative transmembrane domains and an extra-membrane disordered loop, corresponding to the amino acid sequence 126–215. This protein region contains at least three possible metal binding motifs: HxHxHxxD (144–153), HxxHxxEHxD (181–193) and the Glu- and Asp- rich sequence DDEEEDxE (161–168). The corresponding model peptides, protected at their termini (Ac-GPHTHSHFGD-NH_2_, Ac-DDEEEDLE-NH_2_ and Ac-PSHFAHAQEHQDP-NH_2_), have been investigated in order to elucidate the thermodynamic and coordination properties of their Zn^2+^ and Cu^2+^ complexes, with the further aim to identify the most effective metal binding site among the three fragments. Furthermore, we extended the investigation to the peptides Ac-GPHTHAHFGD-NH_2_ and Ac-PAHFAHAQEHQDP-NH_2_, where serine residues have been substituted by alanines in order to check if the presence of a serine residue may favor the displacement of amidic protons by Cu^2+^. In the native Zrt2 protein, the Ac-GPHTHSHFGD-NH_2_ region of the Zrt2 loop has the highest metal binding affinity, showing that three alternated histidines separated by only one residue (-HxHxH-) bind Zn^2+^ and Cu^2+^ more strongly than the region in which three histidines are separated by two and three His residues (-HxxHxxxH- in Ac-PSHFAHAQEHQDP-NH_2_). All studied Zrt2 loop fragments have lower affinity towards Zn^2+^ than the zinc(II) binding site on the Zrt1 transporter; also, all three Zrt2 regions bind Zn^2+^ and Cu^2+^ with comparable affinity below pH 5 and, therefore, may equally contribute to the metal acquisition under the most acidic conditions in which the Zrt2 transporter is expressed.

## 1. Introduction

Trace metals, such as zinc and copper, are essential for the survival of pathogenic microorganisms, including fungi. The acquisition and regulation of these transition metal ions are extremely important to ensure the growth of the cells and the effectiveness of host colonization during infections [1]. The mechanisms put in play by pathogens to obtain the necessary metal nutrients are various and include both active and passive transport through the pathogen cell membranes. In the former case, to make the metal recruitment more efficient, pathogens can synthesize proteins with the specific function of metal scavenger, adapting to the host-mediated metal limitations caused by nutritional immunity response [2].

*Candida albicans* is an opportunistic pathogenic yeast that is part of the human natural microflora. Despite its presence as a commensal organism, it can become very dangerous in immunocompromised individuals: it is the most common cause of urinary tract, genital, and oral yeast infections, and it can develop antimicrobial resistance and lead to severe systemic and potentially life-threatening candidemia (invasive candidiasis) [3].

Many transition metal ions, and in particular zinc, are crucial for the survival and proliferation of *C. albicans* cells in the human organism, and therefore, the mechanisms of metal acquisition can be exploited as a potential target for new antifungal drugs [4]. To ensure proper zinc homeostasis, *C. albicans* relies on two independent protein families of zinc transporters: ZIP (Zrt/Irt-like protein) and ZnT (zinc transporter), which transport zinc from outside the cell into the cytoplasm and vice versa, respectively. Recently, novel insights into the zinc acquisition and regulation processes in *C. albicans* have been obtained and nine putative zinc transporters have been identified. Two of them, Zrt1 and Zrt2, belonging to the ZIP family, were predicted to be localized in the plasma membrane. In particular, Zrt2 proved to be the major zinc importer in *C. albicans,* being crucial to acquire zinc under acidic conditions and cooperating with Zrt1 in the metal uptake at neutral/alkaline pH [5,6].

**Scheme 1:**
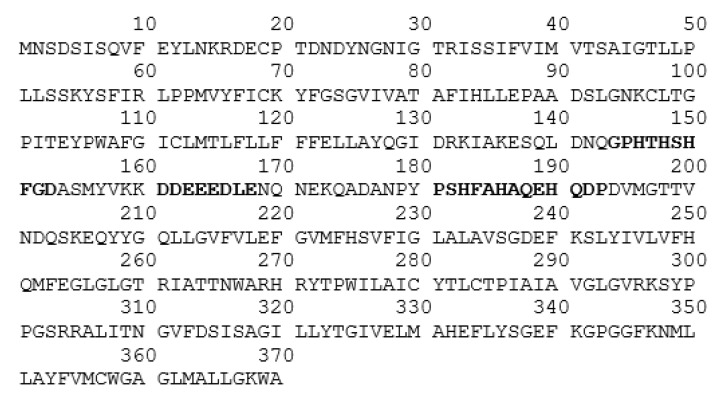
Amino acid sequence of Zrt2 from *C. albicans* [9]. The investigated zinc (and copper) putative binding sites are highlighted in bold.

Since metal acquisition and regulation are important in the physiology and virulence of *C. albicans*, a comprehensive knowledge of the mechanism of metal uptake and transport provides crucial information for the rational design of an effective, specific and selective therapy. Therefore, we focused our studies on the characterization of Zn^2+^-Zrt2 interaction, trying to elucidate the thermodynamics beyond the formation of the metal complexes. Cu^2+^ binding was also investigated, since this cation is a possible competitor for zinc and a necessary nutrient for *C. albicans*. The first step of this work consisted of the identification of the most probable metal binding sites of the protein. Zrt2 from *C. albicans* is composed of 370 amino acids (Scheme 1) and, according to the predicted protein structure provided by AlphaFold software v2.0 [7,8] and UniProt Database (UniProt identifier: A0A1D8PGN5) [9], it contains eight putative transmembrane domains and an extra-membrane, disordered loop corresponding to the amino acid sequence 128–211. The latter protein portion contains at least three possible zinc (and copper) binding motifs: HxHxHxxD (146–153), HxxHxxEHxD (183–192) and a sequence rich in glutamic and aspartic acids, DDEEEDxE (161–168). 

We t**hus** decided to study the thermodynamic and coordination properties of Zn^2+^ and Cu^2+^ complexes with the model peptides corresponding to the identified metal binding sequences: Ac-GPHTHSHFGD-NH_2_ (L1), Ac-PSHFAHAQEHQDP-NH_2_ (L2) and Ac-DDEEEDLE-NH_2_ (L3); we introduced the terminal protection to simulate the behavior of the native fragment within the protein. According to previous studies, the presence of a serine residue in the peptide sequence may favor the displacement of amidic protons by the copper ion, affecting the complex formation ability of the investigated systems [10]. Therefore, in order to verify this property, we also studied the Ser-to-Ala mutants of the wild-type peptides: Ac-GPHTHAHFGD-NH_2_ (L1_S6A) and Ac-PAHFAHAQEHQDP-NH_2_ (L2_S2A).

## 2. Materials and Methods

### 2.1. Materials

All peptides (L1, L1_S6A, L2, L2_S2A, L3) were purchased from KareBay Biochem (Monmouth Junction, NJ, USA) with a certified purity of 98%. They were used as received. CuCl_2_ and ZnCl_2_ were extra-pure products (Sigma-Aldrich, Saint Louis, MI, USA); the concentrations of their stock solutions were standardized by EDTA titration and periodically checked via ICP-OES. The carbonate-free stock solutions of 0.1 M KOH were prepared by diluting concentrated KOH (Sigma-Aldrich) and then potentiometrically standardized with the primary standard potassium hydrogen phthalate (99.9% purity). All sample solutions were prepared with freshly prepared Milli-Q^®^ water. The HCl and HNO_3_ stock solutions were prepared by diluting concentrated ultra-pure HCl and HNO_3_ (Sigma-Aldrich) and then standardized with KOH. The ionic strength was adjusted to 0.1 M by adding KCl (Sigma-Aldrich). Grade A glassware was employed throughout.

### 2.2. Potentiometry

Stability constants for proton and metal complexes were calculated from pH-metric titration curves registered at *T* = 298 K and ionic strength 0.1 M (KCl). The potentiometric apparatus consisted of an Orion EA 940 pH-meter system provided with a Metrohm 6,0234,100, glass-body, micro combination pH electrode and a dosing system Hamilton MICROLAB 500, equipped with a 0.5 mL microburet. The thermostated glass cell was equipped with a magnetic stirring system, a microburet delivery tube and an inlet–outlet tube for the inert gas. High purity-grade nitrogen was gently blown over the test solution in order to maintain an inert atmosphere. Constant-speed magnetic stirring was applied throughout.

Solutions were titrated with 0.1 M carbonate-free KOH. The electrode was daily calibrated for hydrogen ion concentration by titrating HNO_3_ with alkaline solution under the same experimental conditions as above. The standard potential and the slope of the electrode couple were computed by means of SUPERQUAD [11] and Glee [12] programs. The purities and exact concentrations of the ligand solutions were determined by the Gran method [13]. The HYPERQUAD [14] program was employed for the overall formation constant (*β*) calculations, referred to the following equilibrium equation:

(1) $$pM+qL+rH\;⇆{\;M}_{p}L_{q}H_{r}$$

(charges omitted; *p* is 0 in the case of ligand protonation; *r* can be negative). Step formation constants (*K*_step_) and/or acid dissociation constants (*K*_a_) were also reported. The computed standard deviations (referring to random errors only) were given by the program itself and are shown in parentheses as uncertainties to the last significant figure. Hydrolysis constants for metal ions were taken from the literature and suitably extrapolated for the experimental conditions here employed [15,16]. The distribution and competition diagrams were computed using the HYSS program [17]. In particular, the latter were calculated from the binary speciation models, hypothesizing a solution containing the metal and the various ligands, and assuming that all components compete with each other to form the respective binary complexes, without mixed species formation.

### 2.3. Mass Spectrometry

High-resolution mass spectra were obtained on an LCMS-9030 qTOF Shimadzu (Shimadzu, Kyoto, Japan) device, equipped with a standard ESI source and the Nexera X2 system. The mass spectrometer was operated in the positive and negative ion modes. The instrumental parameters were as follows: scan range m/z 100–2000, nebulizing gas nitrogen, nebulizing gas flow 3.0 L/min, drying gas flow 10 L/min, heating gas flow 10 L/min, interface temperature 300 °C, desolvation line temperature 400 °C, detector voltage 2.02 kV, interface voltage 4.0 kV, collision gas argon, mobile phase (A) H_2_O + 0.1% HCOOH, (B) MeCN + 0.1% HCOOH, mobile phase total flow 0.3 mL/min. The injection volume was optimized depending on the intensity of the signals observed on the mass spectrum within the range of 0.1 to 3 μL. The samples were prepared in a 1:1 methanol-water mixture at a pH value of 7.4. The sample concentration was [ligand]_tot_ = 0.1 M, and M:L molar ratio was 1:1. Data were processed using the ACDLabs Spectrus Processor v2021.1.3 program. A comparison between the obtained experimental signals and the true isotopic pattern calculated using Bruker Compass DataAnalysis 3.4 program enabled an unambiguous confirmation of the elemental composition of the obtained complex (Table S2, Figures S15–S17, Supplementary Materials).

### 2.4. Spectroscopic Measurements

The absorption spectra were recorded on a Varian Cary50 Probe spectrophotometer, in the range 350–900 nm, using a quartz cuvette with an optical path of 1 cm. In order to describe the various species obtained in solution, the observed wavelength of maximum absorption at a given pH value (corresponding to the conditions at which a selected species reaches its maximum of formation in solution) was compared with the expected theoretical λ_max_ value obtained from literature [18,19,20]. Circular dichroism (CD) spectra were recorded on a Jasco J-1500 CD spectrometer in the 200–800 nm range, using a quartz cuvette with an optical path of 1 cm in the visible and near-UV range. Electron paramagnetic resonance (EPR) spectra were recorded in liquid nitrogen on a Bruker ELEXSYS E500 CW-EPR spectrometer at X-band frequency (9.5 GHz) and equipped with an ER 036TM NMR teslameter and an E41 FC frequency counter. Ethylene glycol (30%) was used as a cryoprotectant for EPR measurements. The EPR parameters were analyzed by computer simulation of the experimental spectra using WIN-EPR SIMFONIA software, version 1.2 (Bruker, Billerica, MA, USA). The concentrations of sample solutions used for spectroscopic studies were similar to those employed in the potentiometric experiment. The UV-Vis, CD and EPR spectroscopic parameters were calculated from the spectra obtained at the pH values corresponding to the maximum concentration of each species, based on distribution diagrams.

## 3. Results and Discussion

By means of titrimetric and spectroscopic techniques, the speciation models for the studied peptides were obtained. The protonation constants are reported in Table 1, while the complex-formation constants are reported in Table 2 together with the most probable coordination environment for the formed species.

### 3.1. Ligand Protonation

All the investigated peptides are protected at their N- and C-termini by acetylation and amidation, respectively. The peptide acid–base behavior depends, therefore, on the properties of the amino acid side-chains. In the case of peptides L1 and L1_S6A, the protonation sites correspond to 3 alternated histidine residues and one aspartic acid; peptides L2 and L2_S2A contain 3 histidines, one glutamic acid and one aspartic acid; peptide L3 is instead characterized by only acidic residues: 3 aspartic acids and 4 glutamic acids.

The Asp residues had the lowest log*K* values, which varied between 2.73 and 3.92, depending on the primary structure of the peptide and its charge. The obtained protonation constants for Glu residues ranged instead from 4.60 to 5.92, in good agreement with the expectations based on amino acid side-chain protonation equilibria in similar systems [21]; the highest log*K* values of Glu (4.80, 5.23 and 5.92) were obtained for the H_3_(L3)^4−^, H_2_(L3)^5−^ and H(L3)^6−^ species, where the peptide was highly negatively charged due to the other deprotonated acidic residues. The protonation of His residues, which were also the most basic residues in the investigated peptides, occurred with log*K* values ranging from 5.39 to 7.39. On the other hand, the amidic protons of the peptide backbone could not be spontaneously released in the pH range explored by potentiometry, since they were very weak acids (p*K*_a_ ≈ 15) [20], but they could be displaced by Cu^2+^ at a mildly acidic/neutral pH value, to form a coordination bond.

### 3.2. Copper(II) Complexes

**Figure 1:**
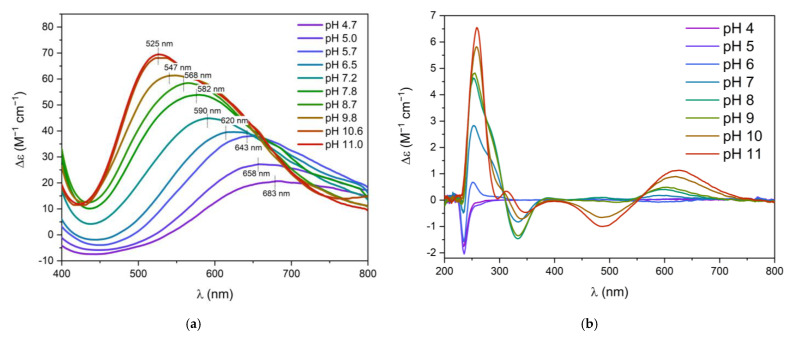
(**a**) Vis absorption spectra and (**b**) CD spectra of Cu^2+^ complexes with L1 at different pH values; M:L ratio 0.8:1. C_M_ = 0.63 × 10^–3^ M, optical path 1 cm. The wavelength of maximum absorption is reported for each Vis absorption spectrum.

The equilibrium constants (Table 2), the MS spectra and the spectroscopic data from UV-Vis, CD and EPR measurements suggest the formation of only mononuclear complexes. From the species distribution diagram depicted in Figure S1 (Supplementary Materials) we observed that copper ion interacts with peptide L1 starting from pH 3, most likely by means of 2 histidine residues (the [CuHL]^2+^ complex), as suggested by the stoichiometry of this species. This complex reaches its maximum of formation at pH 4.7, where the recorded Vis spectrum (Figure 1a) shows a maximum of absorption at λ_max_ = 683 nm, in very good agreement with the expected value for a 2N_Im_ complex (685 nm) [20]. EPR measurements at pH 5 also indicated the presence of a 2N complex (A_//_ = 167; g_//_ = 2.30; g_⊥_ = 2.06, Table S1, Supplementary Materials) [22]. According to the spectroscopic results, the aspartic acid should be not involved in coordination. Increasing the pH value, the first deprotonation step (p*K*_a_ = 5.13) leads to the formation of the [CuL]^+^ species, where the third histidine residue is likely coordinated to the metal ion (3N_Im_ coordination mode). Vis absorption spectrum at pH 6.5, which was characterized by a λ_max_ value of 620 nm, and EPR spectrum at pH 6 (A_//_ = 189; g_//_ = 2.26; g_⊥_ = 2.05) confirmed this coordination hypothesis. The Cu^2+^ interaction with the backbone amides likely begins at around pH 6.5–7. This is highlighted by the circular dichroism spectra recorded at pH 7 (Figure 1b), where the increase in the CD signal intensity can be ascribed to the formation of Cu^2+^-amide bonds. The metal interaction with the peptide backbone, in fact, usually produces a stronger CD absorption, due to the proximity of the *N*-amide donor groups to the peptide chiral centers. An increased square-planar character of the complex geometry is also conceivable, as indicated by the typical shape of CD bands in the visible region [23]. According to the proposed speciation model [CuH_−1_L], [CuH_−2_L]^−^ and [CuH_−3_L]^2^^−^ are the most abundant species found above physiological pH; their coordination sphere should contain up to three deprotonated amides, located in the equatorial plane of the complex, which substitute the imidazole nitrogens as donor groups. This hypothesis was also confirmed by the obtained A_//_ and g_//_ values of the EPR spectra at alkaline conditions (A_//_ = 206; g_//_ = 2.20; g_⊥_ = 2.05). The p*K*_a_ values corresponding to the ionization of the backbone amides were, therefore, 6.85, 6.99 and 9.53 (Table 2).

Ligand L1_S6A, which is the Ser-to-Ala mutant of L1, behaves very similarly to the wild-type ligand, although in this case it was possible to detect the [CuH_2_L]^3+^ species under the most acidic conditions (Table 2, Figure S2, Supplementary Materials). In this first detected complex, one histidine residue should be bound to the copper ion, possibly together with the aspartic acid side-chain (N_Im_, COO^−^). With respect to L1, the interaction with the acidic residue may stabilize this species and make it detectable, although it is quickly substituted by the monoprotonated [CuHL]^2+^ complex. This latter species forms with p*K*_a_ = 4.27, likely corresponding to the binding of a second histidine and, therefore, to the formation of a (2N_Im_, COO^−^) complex. The obtained wavelength of maximum absorption at pH 5 was 654 nm (Figure S6a SI), which confirmed the interaction with a carboxyl donor group (expected λ_max_ = 661 nm). The next deprotonation step (p*K*_a_ = 5.32) led to the formation of a (3N_Im_) complex (obtained λ_max_ = 625 nm, expected λ_max_ = 627 nm), as in the case of L1. EPR data also confirmed the suggested coordination mode for the [CuL]^+^ species, with 3 nitrogen atoms bound to copper (Table S1 SI). Moving to alkaline conditions we observed once again the formation of (3N_Im_, N^−^), (2N_Im_, 2N^−^) and (N_Im_, 3N^−^) complexes, where copper interacted with the amide groups of the peptide chain.

**Figure 2:**
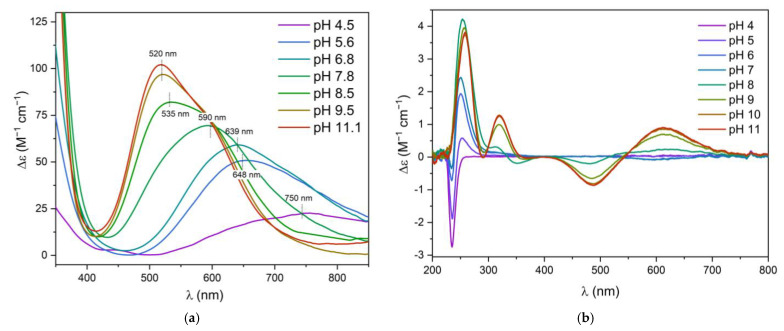
(**a**) Vis absorption spectra and (**b**) CD spectra of Cu^2+^ complexes with L2 at different pH values; M:L ratio 0.8:1. C_M_ = 0.63 × 10^−3^ M, optical path 1 cm. The wavelength of maximum absorption is reported for each Vis absorption spectrum.

In the case of the L2 ligand, Cu^2+^ begins to form complexes at around pH 3 (Figure S3 SI). The first detected complex is [CuH_2_L]^2+^, where one histidine residue should be bound to the metal ion. The acidic residues (Asp and Glu) present in the peptide sequence may also participate in the coordination at acidic pH; however, the employed experimental techniques leave this point questionable. Increasing the pH value, the two histidines deprotonated with p*K*_a_ = 4.71 and 5.46, forming the [CuHL]^+^ and [CuL] species, where the coordination sphere likely involved 2 and 3 histidines, respectively, with a distorted octahedral geometry. In fact, from UV-Vis spectra (Figure 2a) it is possible to clearly distinguish the gradual shift of the λ_max_ value towards higher energies (blue-shift) with the increase in pH. This trend agrees with the hypothesis that, moving to alkaline conditions, a general increase in the number of coordinated nitrogen atoms is observed. EPR results were also consistent with these coordination hypotheses, since at pH 5 and 7 where [CuHL]^+^ and [CuL] are the major species in solution, we obtained the following EPR parameters, which were in good agreement with the theoretical expectations: A_//_ = 163, g_//_ = 2.30, g_⊥_ = 2.06 (pH 5) and A_//_ = 175, g_//_ = 2.27, g_⊥_ = 2.06 (pH 7) [22]. Moving to alkaline conditions, the CD spectra reported in Figure 2b show an increasing signal in the visible region, which can be ascribed to the ionization of the backbone amides and to the formation of Cu^2+^ complexes where *N*-amides occupy the equatorial position of the coordination sphere. It is in fact possible to distinguish two main species, [CuH_−2_L]^–^ and [CuH_−3_L]^2–^, where, according to the obtained spectroscopic results, the coordination modes were (2N_Im_, 2N^−^) (pH 8.5, λ_max_ = 535 nm) and (N_Im_, 3N^–^) (pH 9–11, λ_max_ = 525 nm). The Ser-to-Ala substitution did not decisively affect the coordination properties of the peptide analogue L2_S2A with respect to L2, and the above considerations remain valid also for this system (see Table 2 and Figures S4 and S7, Supplementary Materials).

**Figure 3:**
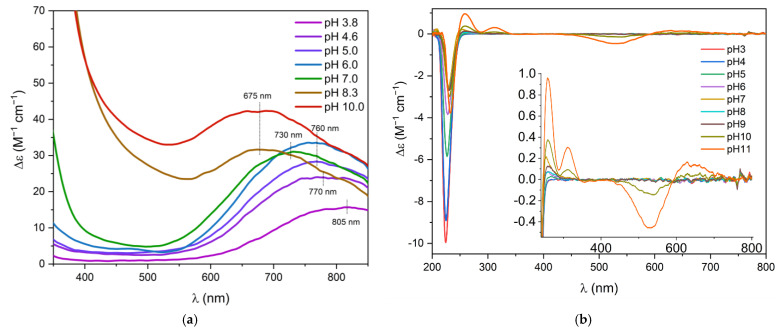
(**a**) Vis absorption spectra and (**b**) CD spectra of Cu^2+^ complexes with L3 at different pH values; M:L ratio 0.8:1. C_M_ = 0.56 × 10^−3^ M, optical path 1 cm. The wavelength of maximum absorption is reported for each Vis absorption spectrum.

The copper-chelating ability of peptide L3 is lower than that of the ligands discussed above. The free Cu^2+^ ion was present in solution until pH around 8, although complexes began to form already at pH 2.5, thanks to the interaction with at least two carboxyl groups of the Asp residues, which gave rise to the neutral species, [CuH_5_L] (Figure S5, Supplementary Materials). Increasing the pH value to neutral conditions, the [CuH_4_L]^−^, [CuH_2_L]^3^^−^, [CuHL]^4^^−^ and [CuL]^5^^−^ species were formed. The release of a proton from [CuH_2_L]^3^^−^ and [CuHL]^4^^−^ occurred with p*K*_a_ = 5.24 and 5.98, respectively. These values may correspond merely to the deprotonation of two glutamic acids, which should not directly participate in the complexation. Interestingly, the obtained spectroscopic data suggest that, after the anchored to the carboxylic moieties of the peptide, the Cu^2+^ ion interacted with the backbone amides only at pH > 10 (Figure 3), confirming the formation of the [CuH_−2_L]^7−^ complex where the metal ion likely coordinates two N-amides. Moreover, in this system, copper hydroxo species were present in solution in large extent, dominating in the alkaline pH range (Figure S5, Supplementary Materials).

### 3.3. Zinc(II) Complexes

In the case of zinc complexes, all of the investigated ligands behaved similarly: they formed variously protonated mononuclear species all over the explored pH range. This was also confirmed by ESI-MS results (Table S2, Supplementary Materials). In particular, according to the obtained species distribution diagrams (Figures S8–S12, Supplementary Materials), we could observe that ligands L1, L1_S6A and L2 interacted with zinc starting from pH 4; the first detected species was the monoprotonated species. According to the stoichiometry of the complex, the zinc coordination sphere should involve two imidazole nitrogens: in fact, protonated histidine residues do not spontaneously release the proton under such acidic conditions, unless they interact with the metal ion that can displace it. In principle, the aspartic acid (in the case of L1 and L1_S6A) and/or the glutamic acid (for L2) could also participate in the complexation to form a (2N_Im_, COO^−^) complex. In the case of L2_S2A, instead, the zinc complexation started at pH 3.5 (Figure S11 SI), forming the [ZnH_2_L]^2+^ species, which was likely characterized by a (N_Im_, COO^−^) coordination mode. This species deprotonated with p*K*_a_ = 5.75, corresponding to the binding of the second histidine residue. Despite the slightly different behavior under the most acidic conditions, for all the four peptides L1, L1_S6A, L2 and L2_S2A, the monoprotonated complex was quickly substituted by the [ZnL] species, which dominated at physiological pH. The associated deprotonation step occurred with a p*K*_a_ value ranging from 5.48 to 6.03, which is consistent with the hypothesis of the binding of a third histidine. A tetrahedral coordination geometry was, therefore, expected with a (3N_Im_, O) coordination, where the oxygen atom may be a carboxylic O^−^ or may belong to a coordinated water molecule.

In the case of ligand L3, the coordination sphere was exclusively involved the carboxylic side chains of Asp and Glu; in particular, three main species were detected under acidic and physiological pH: [ZnH_2_L]^3−^, [ZnHL]^4−^ and [ZnL]^5−^. The release of a proton from the first two complexes occurred with p*K*_a_ = 5.2 and 5.5, these two values wre very similar to those obtained from ligand protonation for the H_2_L and HL species, suggesting that the corresponding Glu residues deprotonate without being involved in zinc complexation.

Despite the different coordination sphere of ligand L3 with respect to the other investigated histidine-containing peptides, it is worth noting that in all of these systems, the free zinc ion remained available in solution until pH 9. Further hydrolysis steps were then observed at alkaline conditions with all five peptides.

### 3.4. A Qualitative Evaluation of Zrt2 Metal-Binding Ability

According to the obtained results, the Ser-to-Ala substitution does not decisively affect the coordination properties of the peptides L1_S6A and L2_S2A with respect to the corresponding native peptides L1 and L2. Nonetheless, the role played by the serine residue in metal complexation can be qualitatively evaluated by means of competition diagrams where each couple of analogues is considered. Such diagrams are based on the calculated stability constants and represent a simulation of solutions containing equimolar concentrations of the metal and the chosen ligands, assuming that all the peptides compete for the metal recruitment and that they form only the binary complexes described in the speciation models.

**Figure 4:**
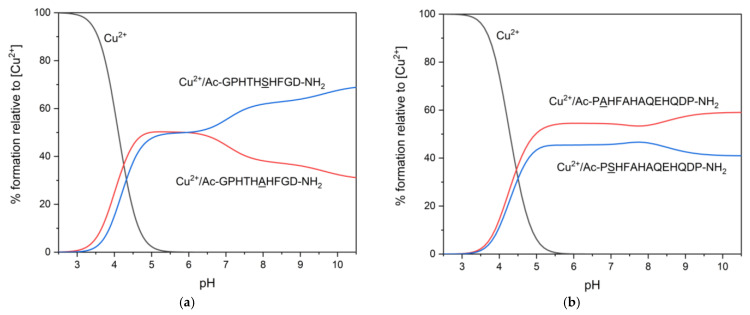
Competition plots for a solution containing equimolar concentrations (1 ×∙10^−3^ M) of (**a**) L1, L1_S6A and Cu^2+^; (**b**) L2, L2_S2A and Cu^2+^.

In the case of L1 and L1_S6A, we could observe, from the competition plot reported in Figure 4a, that the presence of serine stabilized copper complexes above pH 6.5, i.e., when the metal ion began to interact with backbone amides. Although this behavior is not fully elucidated, it quite frequently occurs in Cu^2+^ complexes with peptides, and it is also experimentally verified by several other systems studied in the same or similar conditions [10,24,25]. Therefore, we suggest a possible electronic effect of serines adjacent to the donor residues, that makes the amidic proton displacement more favorable. Apparently, an exception is represented by the two analogues L2 and L2_S2A, where this serine-associated trend was not respected (Figure 4b). These results can be explained assuming that the coordinated amides are located at the C-terminal portion of the peptide, i.e., rather distant from the serine in position 2. In such a case, the Ser/Ala substitution would not affect the binding ability. In fact, the structural constraints due to the presence of a proline residue in position 1 may hinder the amide coordination in the N-terminal domain [26]. Zinc complexes were also compared (Figures S13 and S14, Supplementary Materials), but in this case, the effect due to the presence of the serine residue was negligible. This result can be ascribed to the fact that the amide groups do not interact with Zn^2+^. The absence of a “serine effect” in the case of zinc can be taken as a confirmation of the influence of the Ser residues on the amide deprotonation.

**Figure 5:**
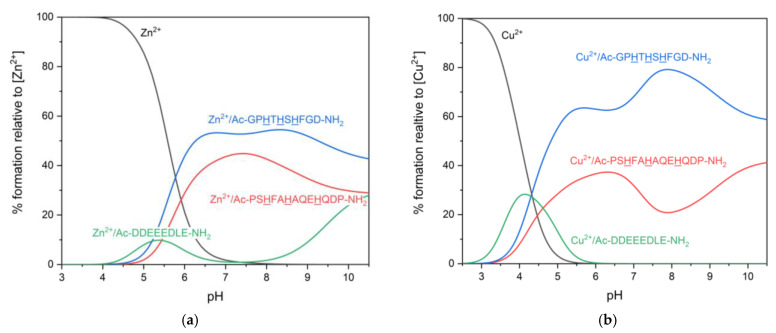
Competition plots for a solution containing equimolar concentrations (1 ×∙10^−3^ M) of L1, L2, L3 and (**a**) Zn^2+^; (**b**) Cu^2+^.

A comparison of the three native peptides L1, L2 and L3 allows us to make speculations on the effectiveness of the identified metal-binding sites in Zrt2 protein. The competition diagrams depicted in Figure 5 show that the alternated histidyl-tag HxHxH (three histidines separated by one residue) was a more effective metal binding motif for both zinc and copper ions, all over the investigated pH range, with respect to the HxxHxxxH sequence, where the three histidines were instead separated by two and three residues. The absence of histidines in the peptide sequence—in the case of L3 containing only Asp and Glu residues—makes the formation of zinc and copper complexes more challenging, and the resulting systems are generally less stable above pH 5. Nonetheless, under the most acidic conditions, the metal binding affinity of the three ligands was comparable.

The stabilities of the complexes formed by L1, L1_S6A, L2, L2_S2A and L3 can be also compared through the parameters reported in Table 3, which give an overall estimation of the metal binding affinity for each peptide at pH 7.4. These parameters take into account all the interactions between the metal ion and the ligand, according to the obtained speciation model, and allow further comparisons with similar biological systems. *K*_d_ (the dissociation constant, expressed as molarity) is referred to a generic equilibrium: ML = M + L (charges omitted) and it is numerically equal to the concentration of the free metal ion when the ligand is half complexed and half not; this means that a smaller value is indicative of greater stability [27]. Similarly, the pM value gives an estimation of the ligand effectiveness, as it is calculated as the negative logarithm of the free metal concentration (−log[M]_free_), under given experimental conditions ([M]_tot_ = 1 × 10^−6^ M, [L]_tot_ = 1 × 10^−5^ M) [28]. The parameter pL_0.5_ was recently introduced to evaluate the metal-ligand affinity on the basis of all the possible competing systems in solution (assuming their speciation models are known) and is defined as the quantity of ligand required to bind 50% of the metal present in traces (typically [M]_tot_ = 1 × 10^−12^ M) [28,29]. The obtained values were in perfect agreement with the competition diagrams (Figure 4, Figure 5, Figures S13 and S14, Supplementary Materials) and designated copper complexes with ligand L1 as the most stable, while the Ser-to-Ala mutant L1_S6A displayed the highest Zn^2+^ binding ability. The effectiveness of the alternated histidyl-tag HxHxH was once again confirmed.

**Figure 6:**
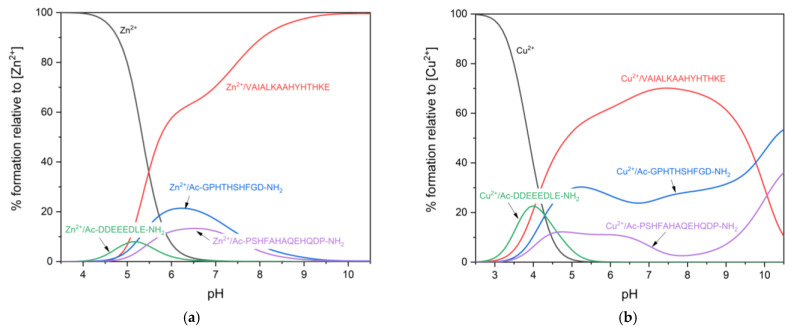
Competition plots for a solution containing equimolar concentrations (1 ×∙10^−3^ M) of VAIALKAAHYHTHKE (calcitermin), L1, L2, L3 (fragments from Zrt2) and (**a**) Zn^2+^; (**b**) Cu^2+^.

To better understand the biological role of the Zrt2 protein, we compared its metal-binding sites with the human antimicrobial peptide calcitermin (VAIALKAAHYHTHKE). Calcitermin is an antimicrobial peptide found in human airways, which corresponds to the cleavage product of the human protein calgranulin C at the carboxyl-terminus. Its antimicrobial activity is enhanced by acidic pH and by the presence of zinc or copper ions [30,31], thus suggesting a possible role of calcitermin in the nutritional immunity process. Furthermore, calcitermin contains three evolutionarily conserved alternated histidines (HxHxH), which can act as metal binding sites under acidic condition. Since only the metal complexes of calcitermin proved to be active against *C. albicans* (minimal inhibitory concentration at pH 5.4 = 1 μg × mL^−1^), and not calcitermin itself, we wanted to verify if this antimicrobial activity was correlated to the effectiveness of calcitermin in binding metal ions. We compared these two chelating systems (calcitermin and the metal-binding sites of Zrt2) (Figure 6), which may potentially compete for metal acquisition during infections, and according to our thermodynamic results, we observed that calcitermin forms more stable complexes than each binding domain of Zrt2, thus suggesting that it is able to withhold metal micronutrients from the surrounding environment. Such results were also confirmed by the *K*_d_ values calculated for Zn^2+^ and Cu^2+^ complexes with calcitermin at pH 7.4 (3.07·× 10^−7^ and 1.31 ×·10^−10^, respectively) [31].

**Figure 7:**
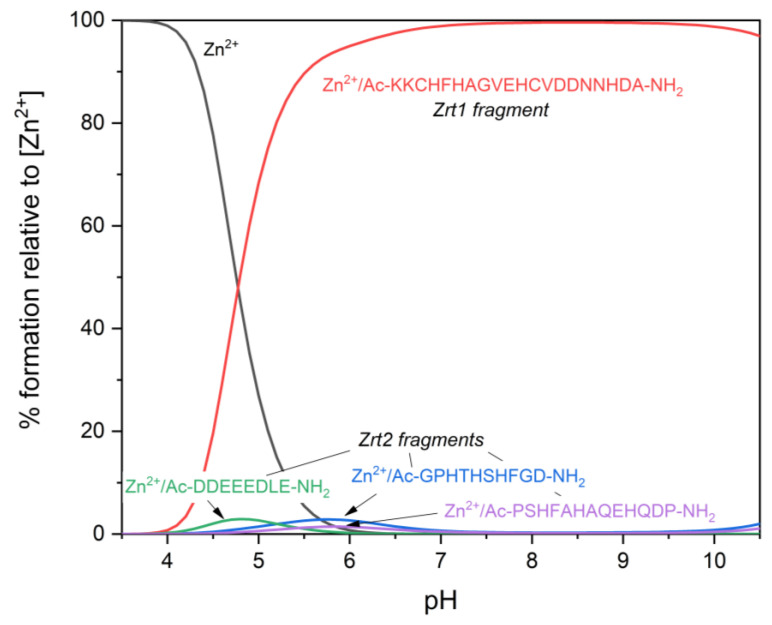
Competition plots for a solution containing equimolar concentrations (1 ×∙10^−3^ M) of Ac-KKCHFHAGVEHCVDDNNHDA-NH_2_ (fragment from Zrt1), L1, L2, L3 (fragments from Zrt2) and Zn^2+^.

As previously mentioned, *C. albicans* can express two main zinc transporters of the ZIP family: Zrt1 and Zrt2. Although they are both involved in zinc acquisition, the pathways of action of these two proteins are slightly different. The main role of Zrt1 is to interact with the zincophore Pra1, which delivers its bound zinc to the transporter protein [32,33]. Zrt2 is instead classified as a low-affinity zinc transporter [9] and is essential for zinc uptake under acidic conditions [5]. A comparison between the zinc binding sites of Zrt1 and Zrt2 is shown in Figure 7. The peptide Ac-KKCHFHAGVEHCVDDNNHDA-NH_2_ has been previously identified as an effective zinc binding site of Zrt1 [34]; therefore, it was chosen to represent the Zrt1 zinc chelating ability. According to the competition diagram, Zrt1 displays a higher metal binding affinity for zinc than Zrt2. This is not completely surprising if we consider the different biological roles played by these two proteins. Zrt1 oversees Zn^2+^ recruitment by interacting with Pra1, which catches zinc from the host environment and must, therefore, exhibit high zinc affinity to compete with the host antimicrobial proteins (e.g., calprotectin). On the other hand, Zrt2 does not have to contend with high-affinity zincophores, but, for instance, it rather may be helped in zinc recruitment by the Sap6 protein. It is a secreted aspartyl protease involved in zinc acquisition, which can increase the metal local concentration and act as a zinc “magnet”, facilitating metal internalization by membrane transporters [6,35].

## 4. Conclusions

**Figure 8:**
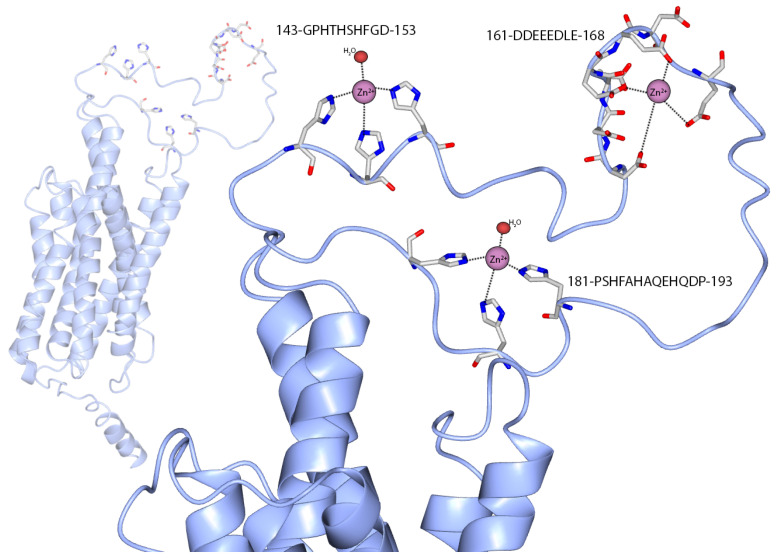
Representation of Zn^2+^ complexes with Zrt2 protein according to the obtained metal coordination modes at acidic/physiological pH. Each fragment can act as an independent metal binding site. The figure was generated using CCP4mg [36]. The 3D structure of Zrt2 was predicted using AlphaFold [7].

The zinc transporter Zrt2 contains three possible metal binding sites located in the extramembrane loop between amino acid residues 140 and 200. The corresponding three studied peptides proved to be excellent ligands for Zn^2+^ and Cu^2+^ ions, although from the obtained results, they are not expected to equally contribute to the metal binding. In fact, according to the competition diagrams shown in Figure 5, which were calculated on the basis of the thermodynamic constants of each binary system, in the native Zrt2 protein, the Ac-GPHTHSHFGD-NH_2_ region should exhibit the highest metal binding affinity. It contains three alternated histidines separated by only one residue (-HxHxH-). The Ac-PSHFAHAQEHQDP-NH_2_ region, which contains three histidines separated by two and three residues (-HxxHxxxH-), shows moderately lower Zn^2+^ and Cu^2+^ affinity. The alternated sequence of Ac-GPHTHSHFGD-NH_2_ was confirmed to be more effective above pH around 5. It is also worth noting that the three peptides, including Ac-DDEEEDLE-NH_2_, exhibit a comparable metal binding ability at pH lower than 5–5.5 and, therefore, they may equally contribute to the metal acquisition under the most acidic conditions, in which the Zrt2 transporter is expressed. Moreover, despite the rather modest metal binding capacity of each single peptide at acidic pH, the three Zrt2 metal-binding sites—which are extramembrane loops—can work independently and participate in metal recruitment, triplicating (Figure 8) the capacity of metal binding, while the different metal binding affinities displayed by the studied sequences may modulate the protein activity in a wider pH range.

## Figures and Tables

**Table 1:** Protonation constants for all investigated ligands at *T* = 298 K and *I* = 0.1 M (KCl). Values in parentheses are standard deviations on the last significant figure.

Species	log*β*	log*K*	log*β*	log*K*	Donor
	**Ac-GPHTHSHFGD-NH_2_ (L1)**		**Ac-GPHTHAHFGD-NH_2_ (L1_S6A)**		
HL	6.96(4)	6.96	6.96(4)	6.96	His
H_2_L^+^	13.32(3)	6.36	13.32(3)	6.36	His
H_3_L^2+^	18.71(4)	5.39	18.87(4)	5.55	His
H_4_L^3+^	21.71(4)	3.00	22.79(4)	3.92	Asp
	**Ac-PSHFAHAQEHQDP-NH_2_ (L2)**		**Ac-PAHFAHAQEHQDP-NH_2_ (L2_S2A)**		
HL^−^	7.39(4)	7.39	7.34(5)	7.34	His
H_2_L	13.90(3)	6.50	13.87(3)	6.53	His
H_3_L^+^	19.79(4)	5.89	19.66(4)	5.79	His
H_4_L^2+^	24.08(4)	4.28	23.87(5)	4.21	Glu
H_5_L^3+^	27.23(4)	3.15	26.73(5)	2.86	Asp
	**Ac-DDEEEDLE-NH_2_ (L3)**				
HL^6−^	5.92(4)	5.92			Glu
H_2_L^5−^	11.15(3)	5.23			Glu
H_3_L^4−^	15.95(6)	4.80			Glu
H_4_L^3−^	20.55(3)	4.60			Glu
H_5_L^2−^	24.45(5)	3.90			Asp
H_6_L^−^	28.28(3)	3.83			Asp
H_7_L	31.01(3)	2.73			Asp

**Table 2:** Equilibrium constants and proposed coordination modes for Cu^2+^ and Zn^2+^ complexes at *T* = 298 K and *I* = 0.1 M (KCl). Values in parentheses are standard deviations on the last significant figure.

Species	log*β*	p*K*_a_	Coordination	log*β*	p*K*_a_	Coordination
	**Ac-GPHTHSHFGD-NH_2_ (L1)**			**Ac-GPHTHAHFGD-NH_2_ (L1_S6A)**		
[CuH_2_L]^3+^	−	−	−	17.68(3)	4.27	N_Im_, COO^−^
[CuHL]^2+^	13.21(2)	5.13	2N_Im_	13.42(1)	5.32	2N_Im_, COO^−^
[CuL]^+^	8.08(2)	6.85	3N_Im_	8.10(2)	7.05	3N_Im_
[CuH_−1_L]	1.22(4)	6.99	3N_Im_, N^−^	1.05(3)	7.27	3N_Im_, N^−^
[CuH_−2_L]^−^	−5.77(3)	9.53	2N_Im_, 2N^−^	−6.22(2)	9.80	2N_Im_, 2N^−^
[CuH_−3_L]^2−^	−15.30(4)	−	N_Im_, 3N^−^	−16.02(3)	−	N_Im_, 3N^−^
	**Ac-PSHFAHAQEHQDP-NH_2_ (L2)**			**Ac-PAHFAHAQEHQDP-NH_2_ (L2_S2A)**		
[CuH_2_L]^2+^	18.45(3)	4.71	N_Im_ (COO^−^)	18.36(3)	4.64	N_Im_ (COO^−^)
[CuHL]^+^	13.74(2)	5.46	2N_Im_	13.72(2)	5.33	2N_Im_
[CuL]	8.27(2)		3N_Im_	8.39(2)		3N_Im_
[CuH_−2_L]^2−^	−6.96(2)	8.61	2N_Im_, 2N^−^	−6.91(2)	8.34	2N_Im_, 2N^−^
[CuH_−3_L]^3−^	−15.57(3)		N_Im_, 3N^−^	−15.25(3)		N_Im_, 3N^−^
	**Ac-DDEEEDLE-NH_2_ (L3)**					
[CuH_5_L]	27.62(7)	3.83				
[CuH_4_L]^−^	23.80(5)	−				
[CuH_2_L]^3−^	15.11(4)	5.24				
[CuHL]^4−^	9.87(5)	5.98				
[CuL]^5−^	3.88(4)	−				
[CuH_−2_L]^7−^	−13.7(1)	−	2N^–^			
	**Ac-GPHTHSHFGD-NH_2_ (L1)**			**Ac-GPHTHAHFGD-NH_2_ (L1_S6A)**		
[ZnHL]^2+^	10.48(9)	5.48	2N_Im_ (COO^−^)	10.71(4)	5.54	2N_Im_ (COO^−^)
[ZnL]^+^	5.00(2)	7.51	3N_Im_	5.17(1)	7.44	3N_Im_
[ZnH_−1_L]	−2.51(4)	9.78	3N_Im_	−2.27(2)	9.43	3N_Im_
[ZnH_−2_L]^−^	−12.29(5)		3N_Im_	−11.69(3)		3N_Im_
	**Ac-PSHFAHAQEHQDP-NH_2_ (L2)**			**Ac-PAHFAHAQEHQDP-NH_2_ (L2_S2A)**		
[ZnH_2_L]^2+^	−	−	−	17.1(1)	5.75	N_Im_ (COO^−^)
[ZnHL]^+^	11.22(5)	6.03	2N_Im_ (COO^−^)	11.36(6)	5.90	2N_Im_ (COO^−^)
[ZnL]	5.19(2)	8.04	3N_Im_	5.46(4)	8.02	3N_Im_
[ZnH_−1_L]^−^	−2.85(3)	9.69	3N_Im_	−2.56(5)	9.82	3N_Im_
[ZnH_−2_L]^2−^	−12.54(5)		3N_Im_	−12.38(7)		3N_Im_
	**Ac-DDEEEDLE-NH_2_ (L3)**					
[ZnH_2_L]^3−^	13.3(5)	5.2				
[ZnHL]^4−^	8.1(4)	5.5				
[ZnL]^5−^	2.6(2)	7.3				
[ZnH_−1_L]^6−^	−4.7(1)	7.8				
[ZnH_−2_L]^7−^	−12.5(1)	−				

**Table 3:** Calculated dissociation constants (*K*_d_), pM and pL_0.5_ values for Zn^2+^ and Cu^2+^ complexes with the investigated peptides at pH 7.4.

Peptide	pZn	pCu	*K*_d_ (Zn^2+^)	*K*_d_ (Cu^2+^)	pL_0.5_ (Zn^2+^)	pL_0.5_ (Cu^2+^)
Ac-GPHTHSHFGD-NH_2_	6.34	9.84	7.84 × 10^−6^	8.65 × 10^−10^	5.10	8.89
Ac-GPHTHAHFGD-NH_2_	6.46	9.53	4.88 × 10^−6^	1.77 × 10^−9^	5.30	8.57
Ac-PSHFAHAQEHQDP-NH_2_	6.27	8.87	1.05 × 10^−5^	8.06 × 10^−9^	4.96	7.92
Ac-PAHFAHAQEHQDP-NH_2_	6.43	9.00	5.41 × 10^−6^	5.97 × 10^−9^	5.26	8.05
Ac-DDEEEDLE-NH_2_	6.00	6.02	9.34 × 10^−4^	1.30 × 10^−4^	2.82	3.71

## Data Availability

Data can be provided from the corresponding author upon reasonable request.

## Supplementary Materials

Supplementary information (SI) is available online at https://www.mdpi.com/article/10.3390/biom12010121/s1. Table S1: EPR parameters for Cu^2+^ complexes with the studied peptides at *I* = 0.1 M (KCl) and M:L molar ratio = 0.8:1, C_L_ = 1.00 × 10^−3^ M (0.79 × 10^−3^ M for L3); Table S2: Stoichiometry, molecular formula and average m/z value for the species present in ESI-MS spectra of Cu^2+^ and Zn^2+^ complexes with the studied ligands, L:M molar ratio = 1:1 in water/methanol 50:50 solution; Figure S1: Species distribution diagram of Cu^2+^/Ac-GPHTHSHFGD-NH_2_, M:L ratio 0.8:1, C_M_ = 0.79 × 10^−3^ M; Figure S2: Species distribution diagram of Cu^2+^/Ac-GPHTHAHFGD-NH_2_, M:L ratio 0.8:1, C_M_ = 0.79 × 10^−3^ M; Figure S3: Species distribution diagram of Cu^2+^/Ac-PSHFAHAQEHQDP-NH_2_, M:L ratio 0.8:1, C_M_ = 0.79 × 10^−3^ M; Figure S4: Species distribution diagram of Cu^2+^/Ac-PAHFAHAQEHQDP-NH_2_, M:L ratio 0.8:1, C_M_ = 0.79 × 10^−3^ M; Figure S5: Species distribution diagram of Cu^2+^/Ac-DDEEEDLE-NH_2_, M:L ratio 0.8:1, C_M_ = 0.79 × 10^−3^ M; Figure S6: (a) Vis absorption spectra and (b) CD spectra of Cu^2+^ complexes with L1_S6A at different pH values, M:L ratio 0.8:1, C_M_ = 0.63 × 10^−3^ M, optical path 1 cm; Figure S7: (a) Vis absorption spectra and (b) CD spectra of Cu^2+^ complexes with L2_S2A at different pH values, M:L ratio 0.8:1, C_M_ = 0.63 × 10^−3^ M, optical path 1 cm; Figure S8: Species distribution diagram of Zn^2+^/Ac-GPHTHSHFGD-NH_2_, M:L ratio 0.8:1, C_M_ = 0.79 × 10^−3^ M; Figure S9: Species distribution diagram of Zn^2+^/Ac-GPHTHAHFGD-NH_2_, M:L ratio 0.8:1, C_M_ = 0.79 × 10^−3^ M; Figure S10: Species distribution diagram of Zn^2+^/Ac-PSHFAHAQEHQDP-NH_2_, M:L ratio 0.8:1, C_M_ = 0.79 × 10^−3^ M; Figure S11: Species distribution diagram of Zn^2+^/Ac-PAHFAHAQEHQDP-NH_2_, M:L ratio 0.8:1, C_M_ = 0.79 × 10^−3^ M; Figure S12: Species distribution diagram of Zn^2+^/Ac-DDEEEDLE-NH_2_, M:L ratio 0.8:1, C_M_ = 0.79 × 10^−3^ M; Figure S13: Competition plots for a solution containing equimolar concentrations (1 × 10^−3^ M) of Ac-GPHTHSHFGD-NH_2_, Ac-GPHTHAHFGD-NH_2_ and Zn^2+^; Figure S14: Competition plots for a solution containing equimolar concentrations (1 × 10^−3^ M) of Ac-PSHFAHAQEHQDP-NH_2_, Ac-PAHFAHAQEHQDP-NH_2_ and Zn^2+^; Figure S15: (a) ESI-MS spectrum for Cu^2+^/L1 system at L:M molar ratio=1:1 in MeOH:H_2_O (1:1) mixture solution, (b) comparison of experimental and simulated isotopic pattern of the chosen metal complex [CuH_2_L]^3+^; Figure S16: (a) ESI-MS spectrum for Zn^2+^/L1 system at L:M molar ratio=1:1 in MeOH:H_2_O (1:1) mixture solution, (b) comparison of experimental and simulated isotopic pattern of the chosen metal complex [ZnH_2_L]^3+^; Figure S17: (a) ESI-MS spectrum for Cu^2+^/L2 system at L:M molar ratio=1:1 in MeOH:H_2_O (1:1) mixture solution, (b) comparison of experimental and simulated isotopic pattern of the chosen metal complex [CuH_3_L]^3+^.
