# Supplementary material for: Zn2+ and Cu2+ Binding to the Extramembrane Loop of Zrt2, a Zinc Transporter of Candida albicans
Source: Biomolecules. 2022 Jan 12;12(1):121. doi: 10.3390/biom12010121 (PMC8773511; doi:10.3390/biom12010121)
Supplement: Supplementary file 1 [file biomolecules-12-00121-s001.zip › biomolecules-1537475-supplementary materials.pdf]

Zn^{2+} and Cu^{2+} binding to the extramembrane loop of Zrt2, a zinc transporter of *Candida albicans*

Denise Bellotti,[1,*] Adriana Miller,[2] Magdalena Rowińska-Żyrek [2] and Maurizio Remelli [3]

[1] Department of Environmental and Prevention Sciences, University of Ferrara, Via L. Borsari 46, 44121, Ferrara, Italy; blldns@unife.it
[2] Faculty of Chemistry, University of Wrocław, F. Joliot-Curie 14, 50-383, Wrocław, Poland; magdalena.rowinska-zyrek@chem.uni.wroc.pl
[3] Department of Chemical, Pharmaceutical and Agricultural Sciences, University of Ferrara, Via L. Borsari 46, 44121, Ferrara, Italy; rmm@unife.it

Supplementary Information

Table S1. EPR parameters for Cu^{2+} complexes with the studied peptides at I=0.1 M (KCl) and M:L molar ratio = 0.8:1. C_L = $1.00 \cdot 10^{-3}$ M ($0.79 \cdot 10^{-3}$ M for L5).

pH	L1					L2					L3				
	$A_{//}$ [G] (A_z)	$g_{//}$ (g_z)	$g_\perp$ (g_x=g_y)	MW Frequency [GHz]	Coordination	$A_{//}$ [G] (A_z)	$g_{//}$ (g_z)	$g_\perp$ (g_x=g_y)	MW Frequency [GHz]	Coordination	$A_{//}$ [G] (A_z)	$g_{//}$ (g_z)	$g_\perp$ (g_x=g_y)	MW Frequency [GHz]	Coordination
**3**	125	2.42	2.08	9.58		122	2.41	2.08	9.57		121	2.41	2.08	9.58	
**4**	123	2.41	2.08	9.59		123	2.41	2.08	9.58		121	2.41	2.08	9.58	
**5**	167	2.30	2.06	9.59	2N	168	2.30	2.06	9.58	2N	163	2.30	2.06	9.58	2N
**6**	189	2.26	2.05	9.59	3N	186	2.25	2.05	9.58	3N	170	2.27	2.06	9.58	2N
**7**	201	2.24	2.05	9.58	3N	189	2.25	2.04	9.58	3N	175	2.27	2.06	9.57	3N
**8**	206	2.20	2.05	9.59	4N	198	2.20	2.04	9.58	4N	183	2.23	2.05	9.57	3N
**9**	206	2.20	2.05	9.58	4N	199	2.20	2.03	9.58	4N	200	2.18	2.05	9.58	4N
**10**	206	2.20	2.05	9.58	4N	199	2.20	2.03	9.58	4N	200	2.18	2.05	9.58	4N
**11**	206	2.20	2.05	9.58	4N	199	2.20	2.03	9.58	4N	200	2.18	2.04	9.58	4N

pH		L4					L5				
		$A_{//}$ [G] (A_z)	$g_{//}$ (g_z)	$g_\perp$ (g_x=g_y)	MW Frequency [GHz]	Coordination	$A_{//}$ [G] (A_z)	$g_{//}$ (g_z)	$g_\perp$ (g_x=g_y)	MW Frequency [GHz]	Coordination
**3**		123	2.42	2.08	9.58		122	2.42	2.08	9.58	
**4**		119	2.42	2.08	9.57					9.58	
**5**		167	2.30	2.06	9.57	2N	140	2.36	2.07	9.58	1N
**6**		172	2.28	2.06	9.58	2N	140	2.36	2.07	9.57	1N
**7**		174	2.27	2.05	9.58	3N	140	2.36	2.07	9.57	1N
**8**		176	2.25	2.05	9.57	3N				9.58	
**9**		192	2.19	2.05	9.58	4N				9.58	
**10**		195	2.19	2.05	9.58	4N				9.58	
**11**		195	2.19	2.05	9.58	4N	186	2.24	2.07	9.58	3N

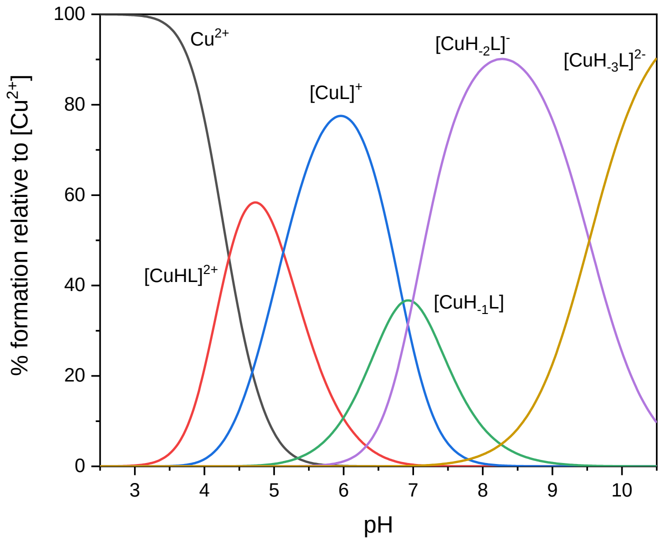

Figure S1. Species distribution diagram of Cu^{2+}/Ac-GPHTHSHFGD-Am; M:L ratio 0.8:1, C_M = $0.79 \cdot 10^{-3}$ M.

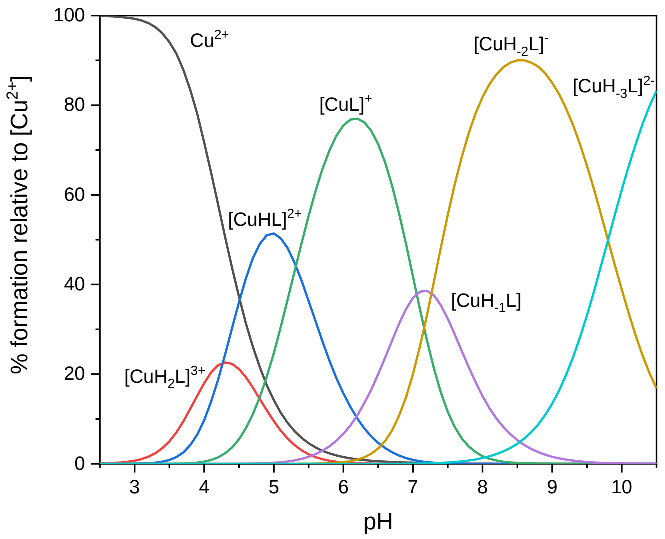

Figure S2. Species distribution diagram of Cu^{2+}/Ac-GPHTHAHFGD-Am; M:L ratio 0.8:1, C_M = $0.79 \cdot 10^{-3}$ M.

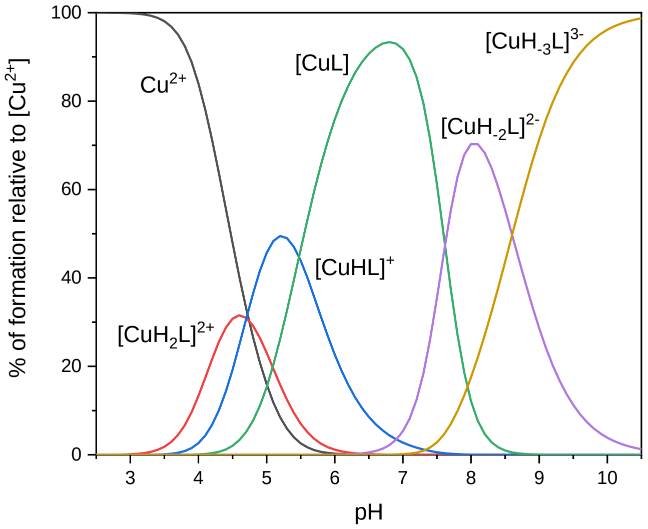

Figure S3. Species distribution diagram of Cu^{2+}/Ac-PSHFAHAQEHQDP-Am; M:L ratio 0.8:1, C_M = $0.79 \cdot 10^{-3}$ M.

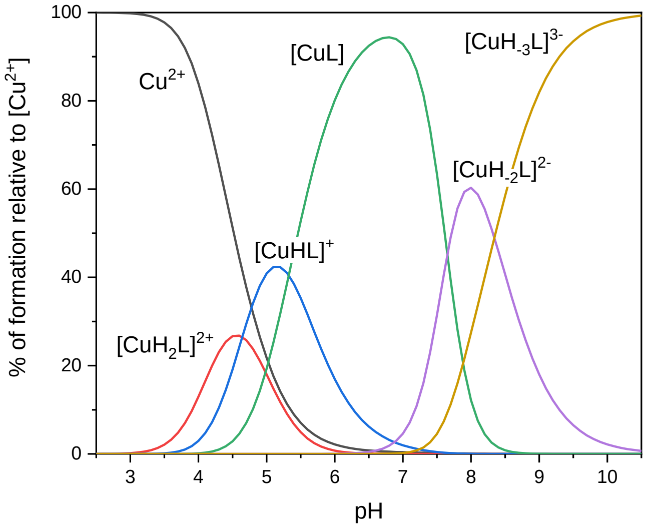

Figure S4. Species distribution diagram of Cu^{2+}/Ac-PAHFAHAQEHQDP-Am; M:L ratio 0.8:1, C_M = $0.79 \cdot 10^{-3}$ M.

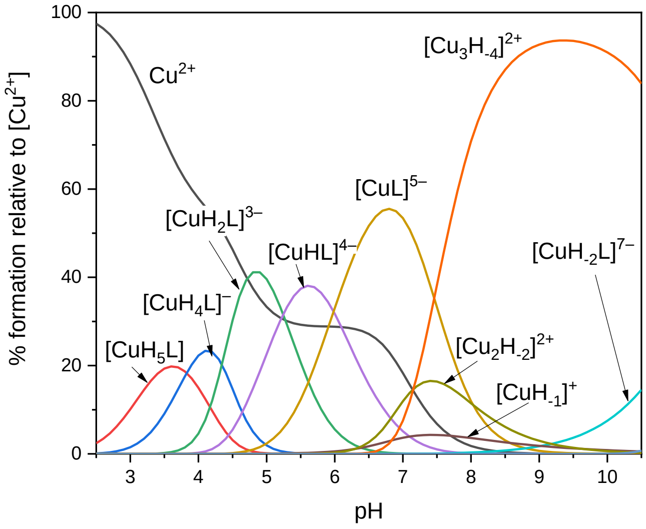

Figure S5. Species distribution diagram of Cu^{2+}/Ac-DDEEEDLE-Am; M:L ratio 0.8:1, C_M = 0.79·10^{-3} M.

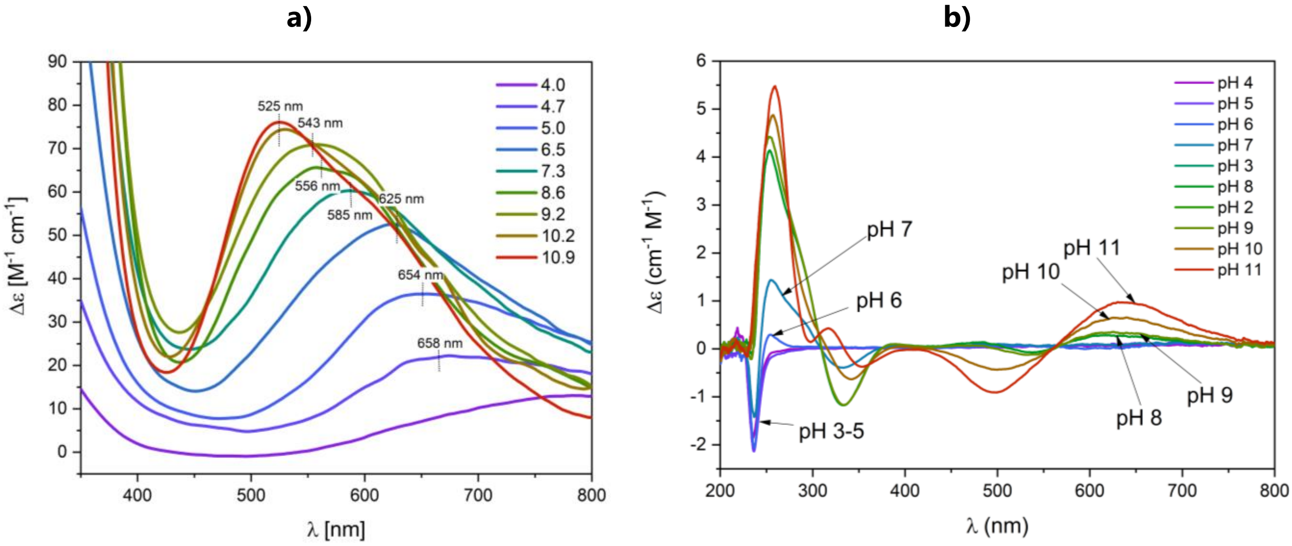

Figure S6. (a) Vis absorption spectra, (b) CD spectra of Cu^{2+}/Ac-GPHTHAHFGD-Am system; M:L ratio 0.9:1. C_M = 0.63·10^{-3} M, optical path 1 cm.

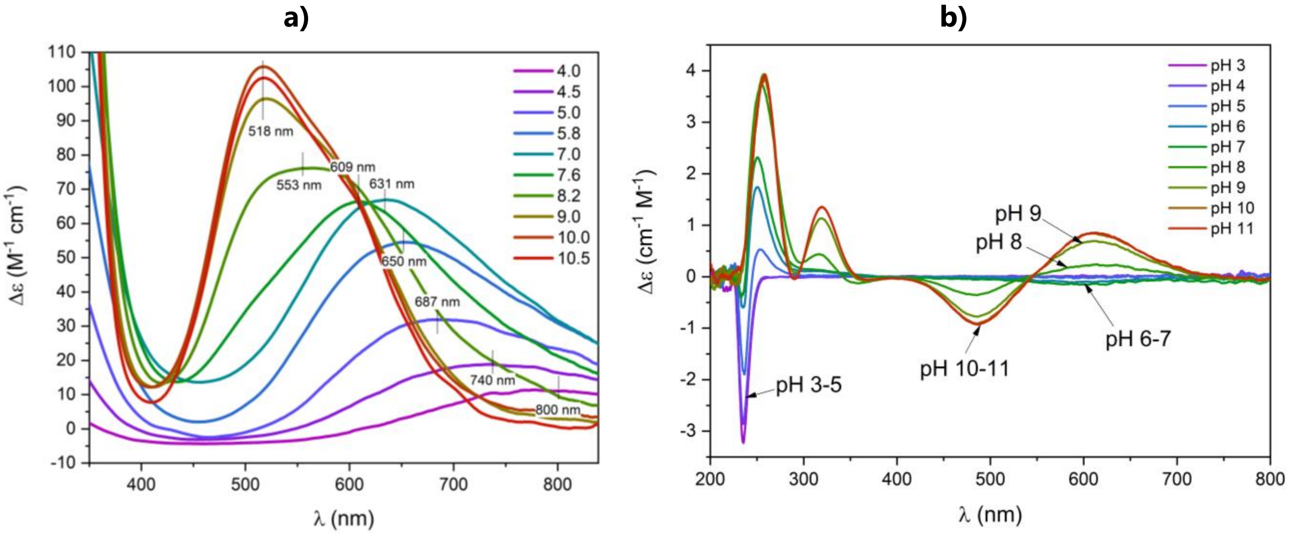

Figure S7. (a) Vis absorption spectra, (b) CD spectra of Cu^{2+}/Ac-PAHFAHAQEHQDP-Am system; M:L ratio 0.9:1. CM = $0.63·10^{-3}$ M, optical path 1 cm.

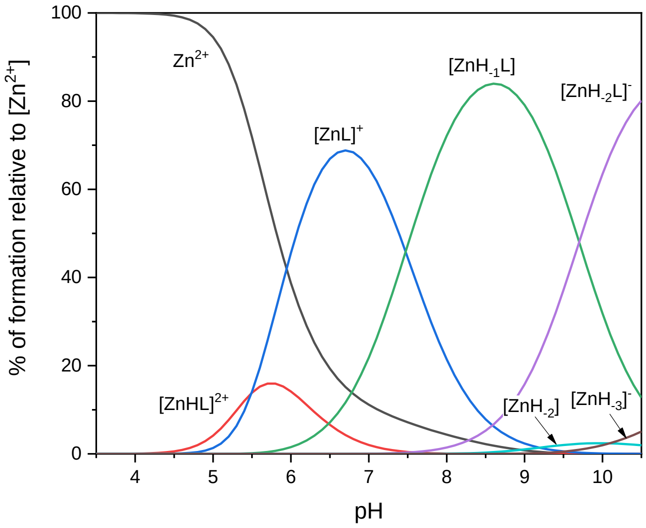

Figure S8. Species distribution diagram of Zn^{2+}/Ac-GPHTHSHFGD-Am; M:L ratio 0.8:1, C_M = $0.79·10^{-3}$ M.

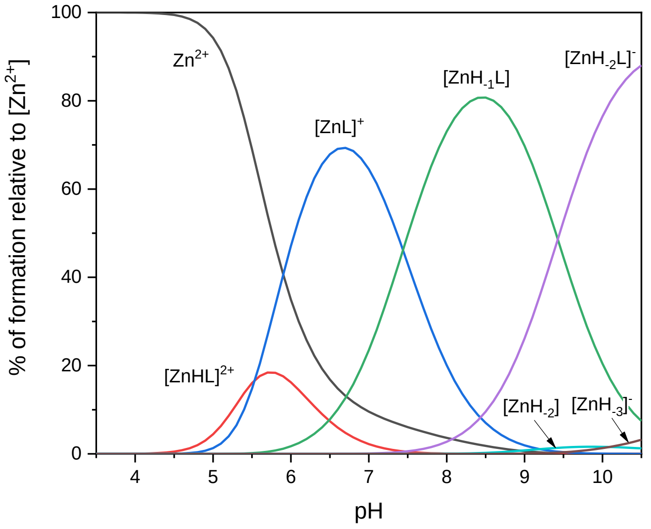

Figure S9. Species distribution diagram of Zn^{2+}/Ac-GPHTHAHFGD-Am; M:L ratio 0.8:1, C_M = $0.79{\cdot}10^{-3}$ M.

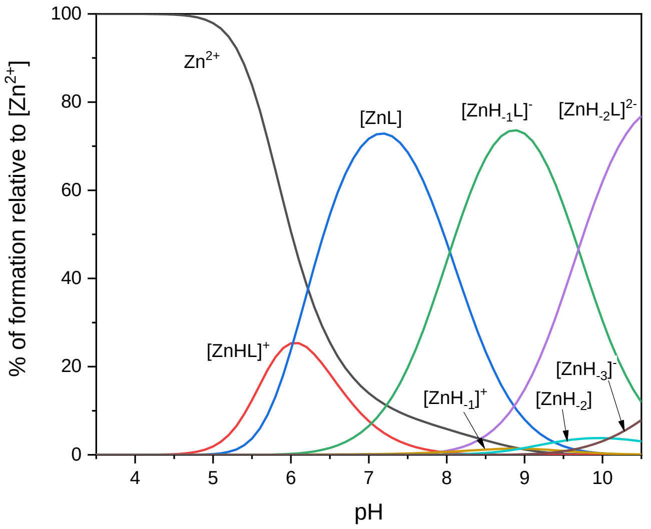

Figure S10. Species distribution diagram of Zn^{2+}/Ac-PSHFAHAQEHQDP-Am; M:L ratio 0.8:1, C_M = $0.79{\cdot}10^{-3}$ M.

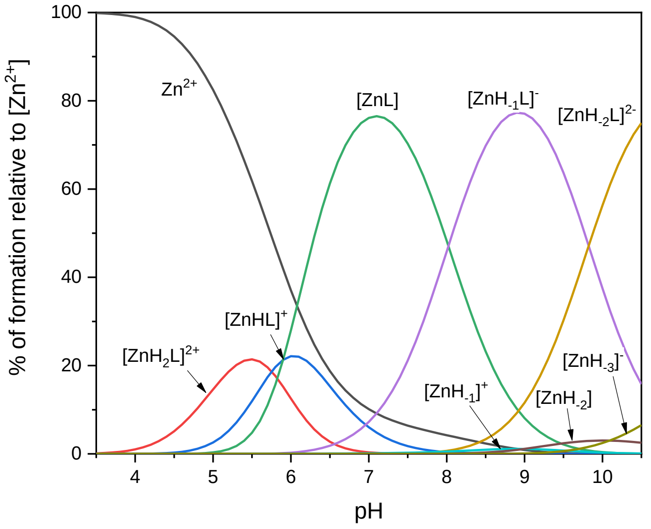

Figure S11. Species distribution diagram of Zn^{2+}/Ac-PAHFAHAQEHQDP-Am; M:L ratio 0.8:1, C_M = $0.79{\cdot}10^{-3}$ M.

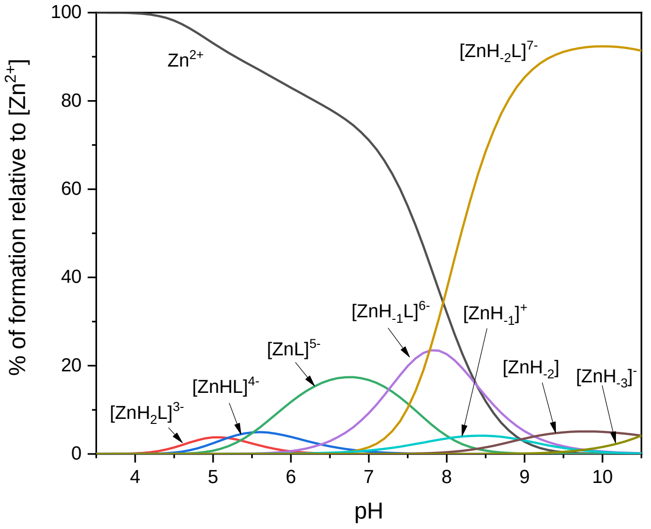

Figure S12. Species distribution diagram of Zn^{2+}/Ac-DDEEEDLE-Am; M:L ratio 0.8:1, C_M = $0.79{\cdot}10^{-3}$ M.

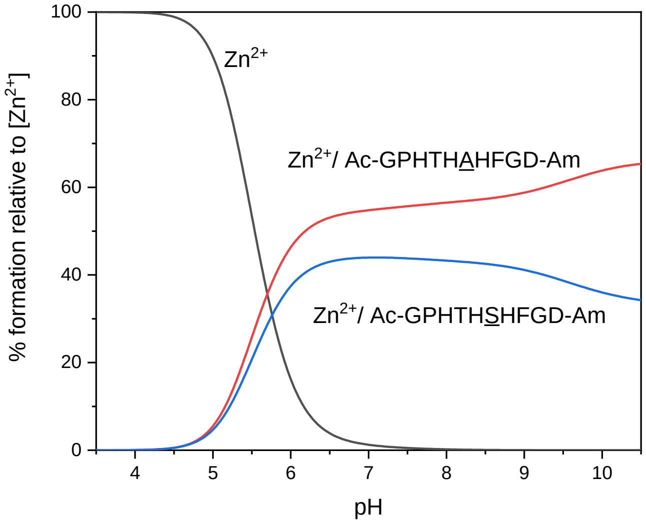

Figure S13. Competition plots for a solution containing equimolar concentrations ($1{\cdot}10^{-3}$ M) of Ac-GPHTHSHFGD-Am, Ac-GPHTHAHFGD-Am and Zn^{2+}.

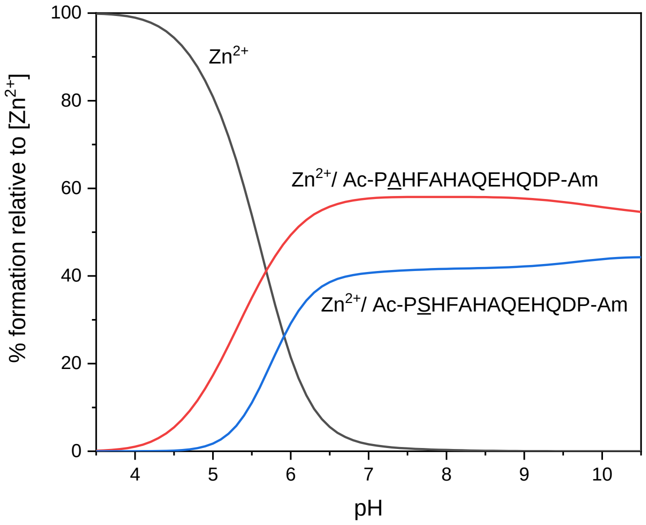

Figure S14. Competition plots for a solution containing equimolar concentrations ($1{\cdot}10^{-3}$ M) of Ac-PSHFAHAQEHQDP-Am, Ac-PAHFAHAQEHQDP-Am and Zn^{2+}.

Table S2. Stoichiometry, molecular formula and average *m/z* value for the species present in ESI-MS spectra of Cu^{2+} and Zn^{2+} complexes with the studied ligands; L:M molar ratio = 1:1 in water/methanol 50:50 solution.

	Species	Formula	Average *m/z*
L1	H_4L^{3+}	C49H68N17O15	378.4
Positive mode	$([H_3L] \cdot Na)^{3+}$	C49H67N17O15Na	385.7
	$([H_3L] \cdot K)^{3+}$	C49H67N17O15K	391.1
	$[CuH_2L]^{3+}$	C49H66N17O15Cu	398.9
	$[ZnH_2L]^{3+}$	C49H66N17O15Zn	399.2
	$([ZnHL] \cdot K)^{3+}$	C49H65N17O15ZnK	412.2
	$([CuL] \cdot NaK)^{3+}$	C49H64N17O15CuNaK	418.9
L2	H_4L^{3+}	C49H68N17O14	373.1
Positive mode	$([H_3L] \cdot Na)^{3+}$	C49H67N17O14Na	380.4
	$([H_3L] \cdot K)^{3+}$	C49H67N17O14K	385.8
	$[CuH_2L]^{3+}$	C49H66N17O14Cu	393.6
	$[ZnH_2L]^{3+}$	C49H66N17O14Zn	394.2
	$([CuHL] \cdot K)^{3+}$	C49H65N17O14CuK	406.3
	$([ZnHL] \cdot K)^{3+}$	C49H65N17O14ZnK	406.9
	$([CuL] \cdot NaK)^{3+}$	C49H64N17O14CuNaK	413.6
	$([ZnL] \cdot K_2)^{3+}$	C49H64N17O14ZnK2	419.6
L3	H_5L^{3+}	C67H95N22O21	514.9
Positive mode	$([H_4L] \cdot Na)^{3+}$	C67H94N22O21Na	522.2
	$([H_4L] \cdot K)^{3+}$	C67H94N22O21K	527.6
	$[CuH_3L]^{3+}$	C67H93N22O21Cu	535.4
	$[ZnH_3L]^{3+}$	C67H93N22O21Zn	536.0
	$([CuH_2L] \cdot K)^{3+}$	C67H92N22O21CuK	548.1
	$([ZnH_2L] \cdot K)^{3+}$	C67H92N22O21ZnK	458.7
	$([CuHL] \cdot NaK)^{3+}$	C67H91N22O21CuNaK	555.4
	$([ZnHL] \cdot NaK)^{3+}$	C67H91N22O21ZnNaK	556.0
L4	H_5L^{3+}	C67H95N22O20	509.5
Positive mode	$([H_4L] \cdot Na)^{3+}$	C67H94N22O20Na	516.9
	$([H_4L] \cdot K)^{3+}$	C67H94N22O20K	522.2
	$[CuH_3L]^{3+}$	C67H93N22O20Cu	530.0
	$[ZnH_3L]^{3+}$	C67H93N22O20Zn	530.7
	$([CuH_2L] \cdot K)^{3+}$	C67H92N22O20CuK	542.7
	$([ZnH_2L] \cdot K)^{3+}$	C67H92N22O20ZnK	543.4
	$([CuHL] \cdot NaK)^{3+}$	C67H91N22O20CuNaK	550.1
L5	H_5L^{2-}	C40H57N9O23	516.0
Negative mode	$([H_4L] \cdot Na)^{2-}$	C40H56N9O23Na	527.0
	$([H_3L] \cdot Na_2)^{2-}$	C40H55N9O23Na2	537.9
	$[CuH_3L]^{2-}$	C40H55N9O23Cu	546.7
	$([H_2L] \cdot Na_3)^{2-}$	C40H54N9O23Na3	548.9
	$([CuH_2L] \cdot Na)^{2-}$	C40H54N9O23NaCu	557.7
	$([ZnH_2L] \cdot Na)^{2-}$	C40H54N9O23ZnNa	558.6
	$([CuH_2L] \cdot K)^{2-}$	C40H54N9O23CuK	565.8
	$([CuHL] \cdot Na_2)^{2-}$	C40H53N9O23CuNa2	568.7

A)

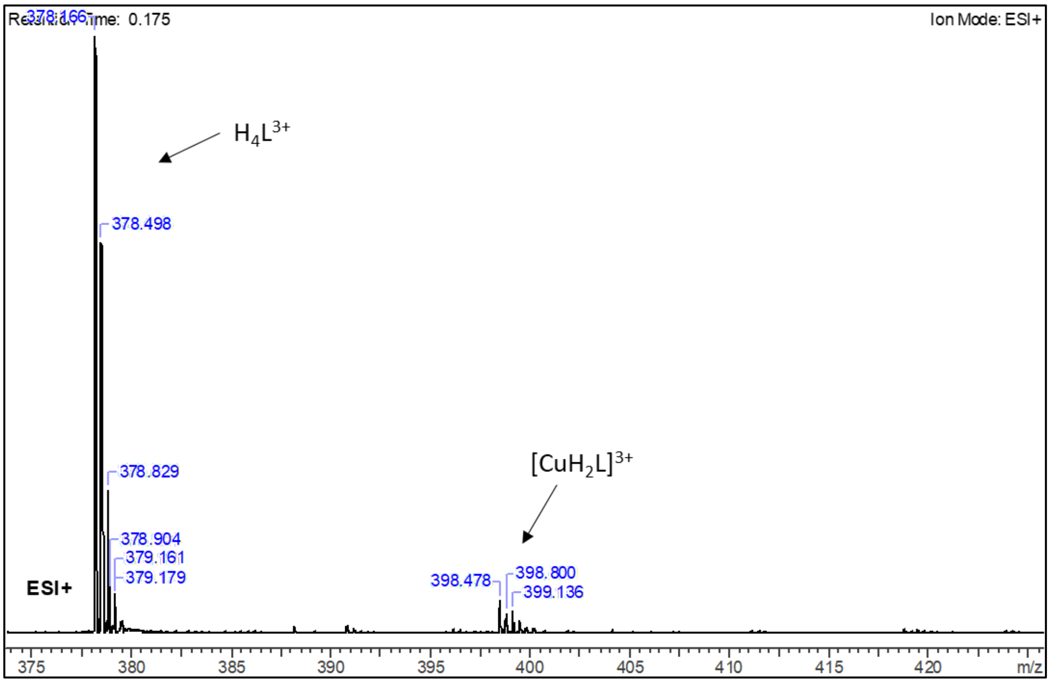

B)

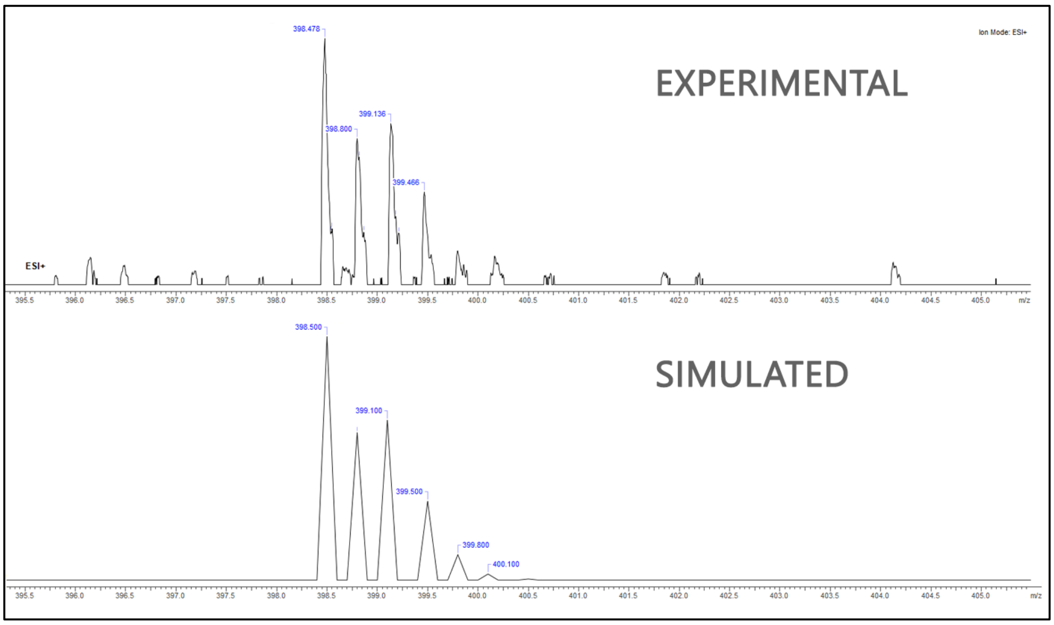

Figure S15. A) ESI-MS spectrum for Cu^{2+}/L1 system at L:M molar ratio=1:1 in MeOH:H_2O (1:1) mixture solution. B) Comparison of experimental and simulated isotopic pattern of the chosen metal complex $[CuH_2L]^{3+}$.

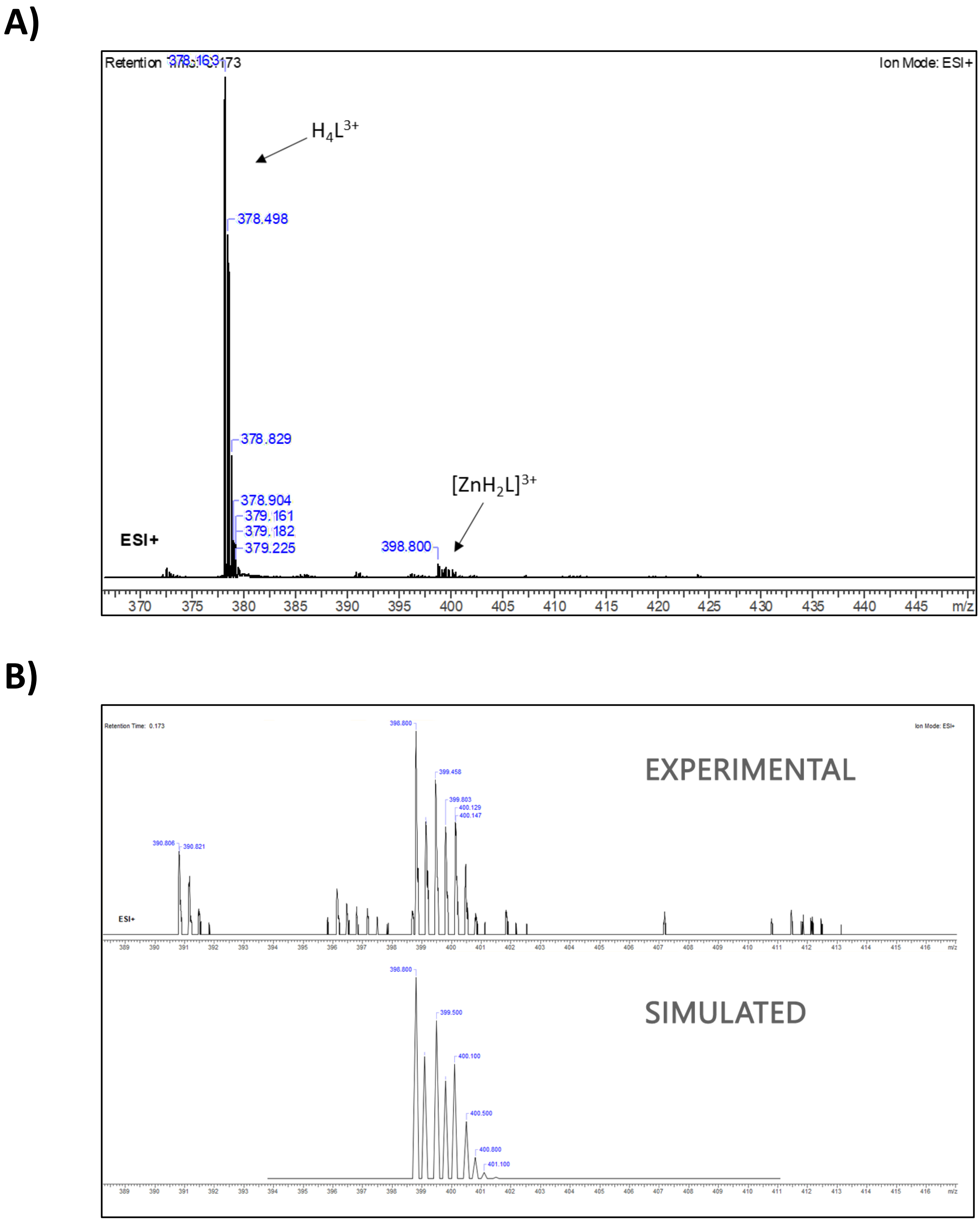

Figure S16. A) ESI-MS spectrum for Zn^{2+}/L1 system at L:M molar ratio=1:1 in MeOH:H_2O (1:1) mixture solution. B) Comparison of experimental and simulated isotopic pattern of the chosen metal complex $[ZnH_2L]^{3+}$.

A)

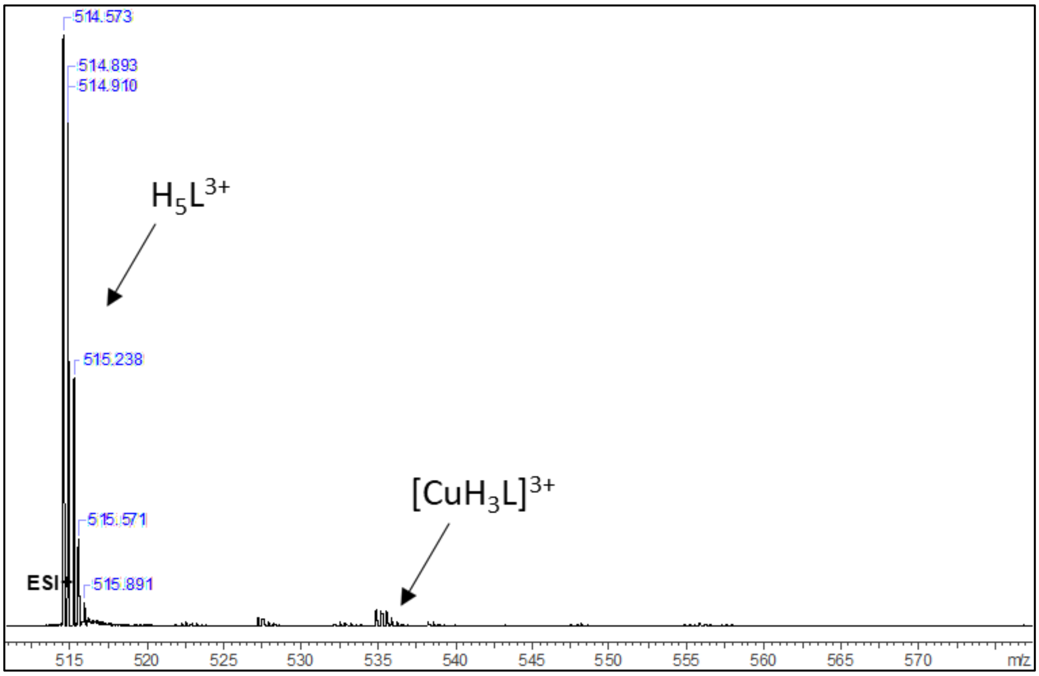

B)

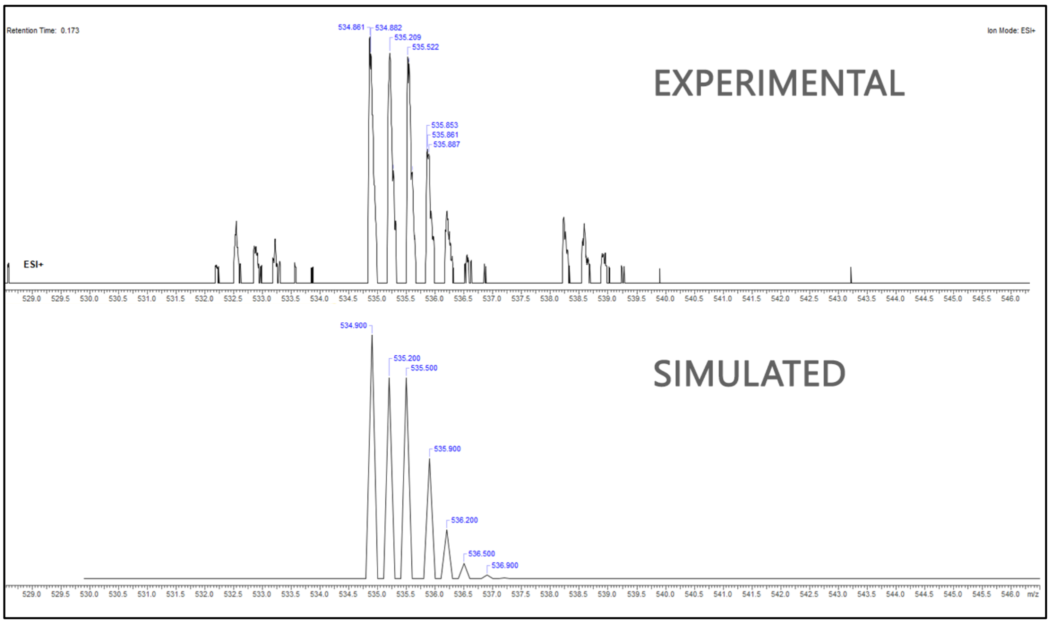

Figure S17. A) ESI-MS spectrum for Cu^{2+}/L3 system at L:M molar ratio=1:1 in MeOH:H_2O (1:1) mixture solution. B) Comparison of experimental and simulated isotopic pattern of the chosen metal complex $[CuH_3L]^{3+}$.
